# Nail bed INJury Assessment Pilot (NINJA-P) study: should the nail plate be replaced or discarded after nail bed repair in children? Study protocol for a pilot randomised controlled trial

**DOI:** 10.1186/s40814-015-0025-z

**Published:** 2015-08-19

**Authors:** Abhilash Jain, Adam Sierakowski, Matthew D. Gardiner, David Beard, Jonathan Cook, Cushla Cooper, Aina Greig

**Affiliations:** 1 Kennedy Institute of Rheumatology, NDORMS, University of Oxford, Oxford, UK; 2 Imperial College Healthcare NHS Trust, London, UK; 3 Mid Essex Hospitals NHS Trust, Chelmsford, UK; 4 Imperial College London, London, UK; 5 Royal College of Surgeons Surgical Intervention Trials Unit, NDORMS, Oxford, UK; 6 Centre for Statistics in Medicine, Royal College of Surgeons Surgical Intervention Trials Unit, NDORMS, Oxford, UK; 7 Guy’s and St Thomas’ Hospitals NHS Trust, London, UK

**Keywords:** Nail bed, Laceration, Repair, Infection, Pain, Appearance, Randomised controlled trial, Plastic surgery

## Abstract

**Background:**

Nail bed injuries account for the majority of paediatric hand trauma cases. Despite their frequency, controversy remains regarding their treatment. The accepted teaching is to remove the fingernail, repair the underlying nail bed with fine sutures and replace the nail under the nail fold. A recent study by Miranda et al. (Plast Reconst Surg. 129(2):394e-396e, 2012) suggests that replacing the nail is associated with increased complications, in particular post-operative infection. Nail bed INJury Assessment Pilot (NINJA-P) is an external pilot study for a large pragmatic, multicentre, randomised, controlled study (NINJA) to assess whether the nail should be replaced or discarded after nail bed repair in children under the age of 16.

**Methods/design:**

NINJA-P is a randomised pilot study. The participants are patients below 16 years of age who require surgical repair of the nail bed. Eligible patients will be randomised to receive one of two possible interventions. Group 1 will have the nail replaced after nail bed repair, and group 2 will have the nail discarded. The clinical outcome measures include the presence of post-operative complications at 2 weeks and 30 days, the cosmetic appearance of the nail at 4 months and the level of pain experienced by the child at their first dressings change at 2 weeks. In order to inform the design of the main NINJA trial, the following feasibility data will also be recorded: the number of potentially eligible children and the proportion which agree to take part in the study, the proportion of children who received the allocated treatment and reasons for any non-compliance and the proportion of participants with a valid response at each follow-up point. Neither the patient, family members nor treating physicians will be blinded. A replaced nail can take several weeks to fall off once a new nail has grown out. The cosmetic appearance of the nail at 4 months will be assessed by a blinded assessor.

**Discussion:**

The NINJA-P pilot study will inform the design and execution of the NINJA trial, which will assess whether the nail should be replaced or discarded after nail bed repair in children under 16. It will provide randomised comparative evidence for the treatment of this common injury.

**Trial registration:**

First participant randomised: 21/04/2015, UKCRN Portfolio ID: 18516, ISRCTN16571591

## Background

Nail bed injuries are common and account for the majority of paediatric hand trauma cases treated by hand surgery units. The typical patient is a toddler who has caught their finger in a closing door. A single tertiary referral hand surgery unit will on average treat two to three patients with nail bed injuries per day. This translates to between 730 and 1095 patients per year. The tariff for a nail bed repair (figure from Guy’s and St Thomas’ NHS Trust) is £1086, so this activity can be expected to generate between £792,780 and £1,189,170 of income per annum for a NHS Trust and a significant expense to the NHS.

**Fig. 1:**
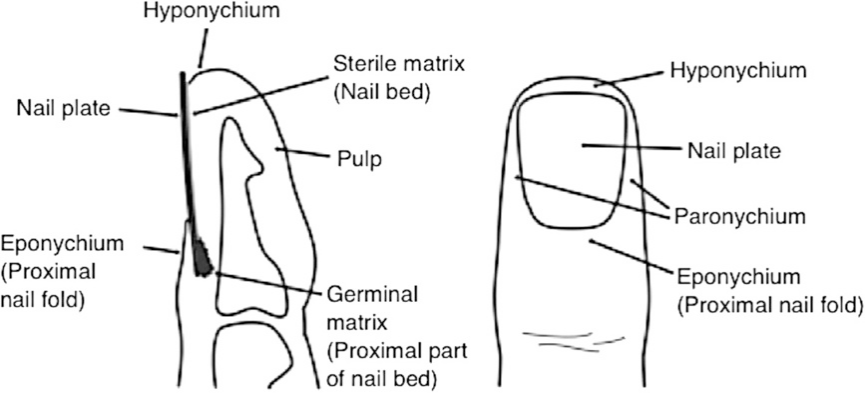
Anatomy of the fingertip

Despite their frequency, controversy remains around the appropriate treatment of nail bed injuries [1, 2]. Without proper treatment, injury to the nail complex has the potential to cause considerable dysfunction and/or deformity [3–7]. The long-accepted teaching has been to remove the nail plate (i.e. the fingernail), repair the underlying matrix (i.e. nail bed) laceration with fine absorbable sutures and replace the nail plate under the eponychium (i.e. proximal nail fold, Fig. 1) [8–11]. The replaced nail has no capacity for re-growth. Instead, as a new nail begins to grow, the replaced nail is gradually pushed out until it becomes loose. The rationale for replacing the nail is that it both protects the nail bed repair and acts as a ‘splint’ by holding open the nail fold and preventing scarring between the nail fold and the nail bed (synechiae). However, there is no evidence that replacing the nail has better results than not replacing it.

A recent study by Miranda et al. suggests that replacing the nail plate is associated with increased complications, in particular, the development of post-operative infection [12]. This retrospective study examined the outcomes of nail bed repairs in 111 children. Overall complications occurred more frequently in the nail replacement group (17.6 %) versus the nail discarded group (5 %) (*p* < 0.001). In particular, the occurrence of delayed wound healing (11.7 versus 3.3 %; *p* = 0.004) and post-operative infection (7.8 versus 0 %; *p* < 0.0001) was significantly greater in the nail replacement group. There were significantly more outpatient visits and a longer overall follow-up period required in the nail replacement group compared to the nail discarded group. The authors advocate simply discarding the nail plate and dressing the repaired nail bed with a simple non-adherent dressing.

The reason for this apparent increase in infective and wound healing complications is thought to be that the replaced nail plate acts as a foreign body to ‘trap’ bacteria and make infection more likely. If this is the case, the simple act of discarding the nail plate could result in a significant reduction in the overall morbidity associated with this procedure, as well as a reduced burden on the NHS associated with repeated follow-up visits, antibiotic courses and, in severe cases, hospital readmission.

Our own background research, including a nationwide survey of orthopaedic and plastic surgeons, has found that the vast majority (96 %) replace the nail plate without great justification. A recent Cochrane review concluded that “there is a lack of evidence from RCTs to inform all key treatment decisions for the management of fingertip entrapment injuries in children” and that “further RCTs are required in this area” [13]. The authors recommended that outcome assessment should be at a minimum of 3 months post-treatment, as this represents the minimum time for a new nail to re-grow.

The Nail bed INJury Assessment (NINJA) study therefore seeks to answer the question “should the nail plate be replaced or discarded after nail bed repair in children, as evaluated by overall complications and appearance of the nail (co-primary outcome measures)?” A consensus meeting was held at the Royal College of Surgeons in February 2014 to discuss the protocol (which included surgeons from across the country along with methodologists). A number of key uncertainties exist regarding the design and conduct of this study including recruitment feasibility, outcome measures and trial pathway appropriateness. This pilot study will assess these and inform the design of the anticipated subsequent multicentre randomised controlled trial (NINJA). A 4-month follow-up time point will be used in the pilot study whereas the main study will require longer term follow-up as it is recognised that subtle improvements in the cosmesis of the nail will occur with subsequent growth cycles.

## Methods/design

### Objectives of the study

NINJA-P is an external pilot study for a large pragmatic, multicentre, randomised, controlled study (NINJA Trial) to assess whether the damaged nail should be replaced or discarded after nail bed repair in children under 16. The primary objective of NINJA-P is to inform the design of the anticipated subsequent main NINJA study specifically in terms of feasibility of recruitment and data collection. In order to achieve this, a range of feasibility measures and clinical outcomes will be collected as listed below.

The feasibility measures are as follows:

1. Number of potentially eligible children
2. Number of patient/parents and guardian’s approached to take part in the study
3. Proportion of children for whom consent was sought which took part in the study
4. Proportion of children who received the allocated treatment and reasons for any non-compliance
5. Proportion of participants with a valid response at each follow-up point (for 4-month time point both overall and only by method of follow-up)

The patient-centred outcome measures are as follows:

1. The presence of post-operative complications at 2 weeks and 30 days
2. The cosmetic appearance of the nail at 4 months
3. The level of pain experienced by the child at their first dressing change at 2 weeks

The secondary study objectives are to inform the design and conduct of the main NINJA trial as follows:

1. Identify any conflicts or areas of concern for the research pathway compared with the existing standard clinical pathway.
2. Assess suitability of outcome measures for children in this setting.
3. Quantify event proportion and variability data to help inform a sample size calculation for main study.

### Recruitment

Trial participants will be prospectively recruited for the trial from the participating hospitals (Guy’s and St Thomas’ Hospitals NHS Trust, London, UK; Mid Essex Hospitals NHS Trust, Chelmsford, UK; Hull and East Yorkshire Hospitals NHS Trust, Hull, UK; Oxford University Hospitals NHS Trust, Oxford, UK). Screening and eligibility assessment will take place when the patient is first seen by a member of the specialist surgical team in the emergency department or paediatric ward of the participating unit. In the majority of cases, this task will be performed by a trainee surgeon (Foundation Year, Core Trainee or Specialist Trainee grade).

The setting in which the patient is first seen will depend on the local protocol at the participating hospital. For example, in some hospitals, the specialty surgeon will be asked to see patients in the emergency department by a referring emergency practitioner. In other hospitals, the specialty surgeon may ask the referring practitioner to send the patient to a paediatric ward, or other assessment area, for review. In either circumstance, it will only be a member of the specialist surgical team who will perform screening and eligibility assessment, not a member of the emergency department or paediatric team. Ultimately, the patient will always be asked to attend a paediatric ward prior to surgery.

The eligibility assessment will require examination of the child’s fingertip with the dressings removed, combined with review of the child’s radiographs, if indicated (e.g. in crush injuries), to check for the presence of a fracture. Parents of eligible patients will be asked by the assessing surgeon if they would like their child to take part in the trial and be provided with the age-appropriate information sheets. It is anticipated that, in most circumstances, consent will be sought by the same person carrying out the eligibility assessment and in the same setting. Depending on the resources of the participating unit, there will be several Good Clinical Practice (GCP)-trained trainees and/or consultant surgeon(s) capable of performing both the eligibility assessment and consent process. In some units, the consent process may be undertaken by a research nurse, after the patient has been deemed eligible for the study by a specialty surgeon. These roles will be recorded in the centre’s delegation log.

### Informed consent

The parent(s) or legal guardian of patients enrolled in the study will need to give informed, written consent preoperatively. Consent will be obtained by individuals who are trained in GCP and consent and who are listed on the delegation log. Written and verbal versions of the participant information and informed consent will be presented to the participants parents/legal guardians detailing no less than the exact nature of the study, what it will involve for the participant, the implications and constraints of the protocol and any risks involved in taking part.

It will be clearly stated that the child is free to withdraw from the study at any time for any reason without prejudice to future care and with no obligation to give the reason for withdrawal. Consent for medical photography will be included as part of the consent process. The patients themselves will also be provided with an information sheet telling them about the study. Separate patient information sheets will be available for the following age groups under 6, 6–10, 11+. If, having had adequate time to make a decision, the parent or legal guardian is happy to give their consent, this will be recorded by means of a signature on a standardised consent form for the study.

### Number of participants

The target sample of size of this study is 60 patients. A study of this size would be sufficient to generate a binary feasibility measure with a 95 % confidence interval width of between 15 and 25 % depending upon the event proportion based upon the Wald interval [14].

### Eligibility criteria

#### Inclusion criteria

- Aged below 16 years of age
- Acute nail bed injury (occurring within 48 h of presentation at trial centre) requiring surgical repair
- Injury-type includes sharp lacerations, stellate lacerations, crush and avulsion injuries of the nail bed, injuries involving the sterile and/or germinal matrix, nail bed injuries with an associated pulp laceration and/or with an associated ‘tuft’ fracture of the distal phalanx
- Patients whose parent or legal guardian consent to their inclusion in the trial and are willing to return for follow-up

#### Exclusion criteria

- Patients aged 16 years or over will be excluded.
- Patients who present with an already infected nail bed injury.
- Patients with underlying nail disease or deformity prior to the injury.
- Patients with an associated distal phalanx fracture requiring fixation with a Kirschner wire. This is considered to be another potential source of infection and therefore a confounding variable.
- Patients with complete amputation of the distal fingertip including all or part of the nail bed, which requires repair as a composite graft or replantation.
- Patients with loss of part or all of the nail bed requiring a nail bed graft or flap reconstruction.
- Non-English speaking individuals.

### Interventions

**Fig. 2:**
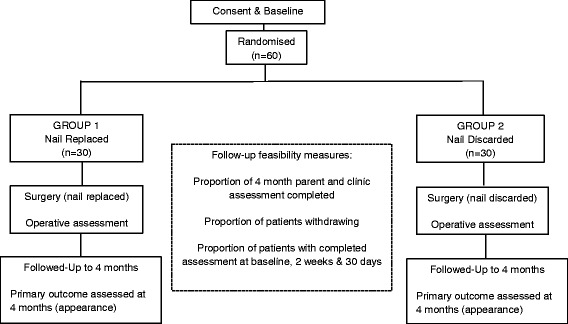
Flow chart of the study

Eligible patients will be randomised to receive one of two possible surgical interventions (Fig. 2). The surgery will be performed by a surgeon adequately trained to be able to undertake a nail bed repair in line with the requirements of the study. This will usually be the surgical trainee assigned to trauma that day or the trauma fellow.

### Intervention 1: replaced nail (group 1)

Group 1 will have the nail plate replaced after nail bed repair. The nail bed repair will be performed after adequate washout and debridement, and the repair completed using 6/0 or 7/0 interrupted sutures. The nail plate will be secured using a figure-of-eight Vicryl Rapide suture, orientated in the longitudinal axis. If the nail plate cannot be replaced in a patient randomised to group 1, for example, if it is too badly damaged, a nail substitute of the operating surgeon’s choice will be used.

### Intervention 2: discarded nail (group 2)

Group 2 will have the nail plate discarded after the nail bed repair. The washout and debridement and suturing procedures will be the same as described for the first group.

This will be a pragmatic study; hence, the following decisions (whichever arm of the study the patient is in) will be left to the discretion of the surgical team responsible for the patient and recorded appropriately:

- The type of anaesthetic used (general or local, or both). No stipulation will be made regarding the type of anaesthetic to be used. In this age group, it is normal for the majority of cases to be performed under general anaesthetic as it is uncommon for a young child to be cooperative enough to tolerate a local anaesthetic procedure.
- Perioperative antibiotics given, if any.
- Type of tourniquet used.
- Type of wash used.

The fingertip will be dressed with the same non-adherent dressing in both groups.

Participants in NINJA-P are additionally being randomised to receive one of two follow-up methods for data collection at 4 months (in a manner similar to a SWAT [15]). Half of the patients will be seen in clinic at 4 months to assess the cosmetic appearance of their fingernail. The other half will be followed up remotely using a postal questionnaire, which will also involve asking parents to take photographs of their child’s repaired fingernail and the contralateral unrepaired nail emailing the photographs to a secure address for assessment.

### Randomisation

A web-based randomisation system will be provided by the Oxford Clinical Trials Research Unit (OCTRU) with allocation stratified by site and formed of random permuted blocks of varying size. Each participant will receive two allocations, one for treatment and one for method of follow-up at 4 months (see below for further details). Randomisation will, ideally, take place when the patient is in the anaesthetic room, just prior to surgery. A member of the local research team, who will not be blinded to the treatment, will complete the randomisation process. This will require secure login to the web-based randomisation system by a computer or handheld device connected to the internet. The computer programme will ask for confirmation that the inclusion criteria have been met and that no exclusion criteria are present. Eligible participants will be assigned a unique trial number, and the programme will inform the research team member of the surgical intervention the participant should undergo and which follow-up method they have been allocated to for the 4-month time point. The designated intervention will be recorded in the case report form (CRF) and, if the person randomising the patient is not the operating surgeon, this information will be relayed to the operating surgeon before the start of the operation.

### Feasibility data collection

The trial coordinator will collate information from screening logs, change of status forms and from the completion of the follow-up questionnaires throughout the trial. Screening log data will provide information regarding the number of potential patients who were approached, the number who decline and any given reasons for this. Change of status forms will inform the study group of reasons patients withdrew, or were withdrawn by their clinician, from their randomised treatment and/or follow up. The trial database will provide information about the completeness of study forms and attendance at follow-up appointments. The coordinator will also monitor and record feedback from patients/parents and participating clinicians in relation to the follow-up schedule and the content of the CRFs and patient questionnaire.

### Baseline assessments and operative assessments

#### Baseline assessment: day of operation, acute hospital setting (emergency department, paediatric ward, operating theatre)

**Fig. 3:**
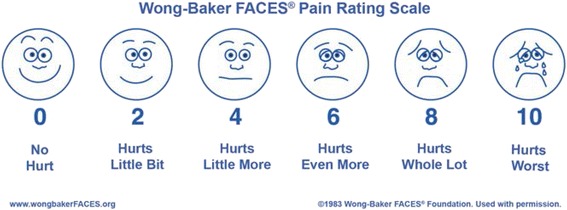
The Wong-Baker FACES pain rating scale

Patient demographics will be recorded on the CRF when the patient is seen by the assessing surgeon on admission in the emergency department or the paediatric ward. Data recorded will include hospital number, date of birth, sex, pre-existing health problems and regular medications, the digit involved, the method of injury and the presence or absence of a fracture (Fig. 3).

#### Operative assessment

At the time of surgery, the operating surgeon will classify the nail bed injury according to the system proposed by Zook et al. [10] and record this on the CRF. Details of the operation carried out, including whether or not the nail was replaced, operative complications, if a nail substitute was used, type of anaesthetic used, any antibiotics given, operative and tourniquet time and the grade of surgeon carrying out the operation will also be recorded. Any procedures which might lead to exclusion of the patient from the study (such as insertion of a Kirschner wire) will also be recorded, and the principal investigator for the site will be informed.

### Follow-up assessments

Participating patients will be required to attend hospital for assessment of patient-centred outcomes at the following time points:

#### 2 weeks, clinic

The dressings from surgery will be removed in clinic by either the surgeon or a nurse. It is not routine practice to give analgesia to children prior to having their dressings removed. However, as this is a pragmatic study, if analgesia is required, this will be recorded on the CRF. The level of pain reported by the patient according to the Wong-Baker FACES pain rating scale [16] will be recorded on the CRF by a clinical research nurse or attending surgeon (Fig. 3). This pain scale has been validated for scoring acute pain in children presenting at the emergency department [17] and is therefore considered an appropriate means of scoring the acute pain associated with removing a dressing from over a wound. The member of the research team recording the pain level will also assess the child’s fingertip for the absence or presence or of any complications (including infection as defined according to Centers for Disease Control (CDC) definition of surgical site infection [18]) and record these on the CRF. Information about any further treatment sought since their operation will also be collated. Complications will be treated as clinically indicated. The fingertip will be re-dressed with a simple non-adherent dressing, either by the surgeon or a nurse.

#### 30 days, clinic

The fingertip will again be assessed by a clinical research nurse or attending surgeon for the presence or absence of any complications. Information about any further treatment sought in between follow-up assessment time points will also be collated. These will be recorded on the CRF. Complications will be treated as clinically indicated.

#### 4 months, clinic and photograph submission

Half of the patients will be seen in clinic at 4 months to assess the cosmetic appearance of their fingernail. The decision as to who will attend the clinic for the 4-month visit and who will just send a photo will have been made at time of randomisation. During the clinic review, a standardised photograph will be taken of the fingernail. This will be taken by a surgeon or clinical research nurse when the child attends clinic using a digital camera. The photograph will include both the affected fingernail and contralateral normal fingernail for comparison. An example of a standardised photograph, with the format required, will be available in the clinic for guidance.

**Fig. 4:**
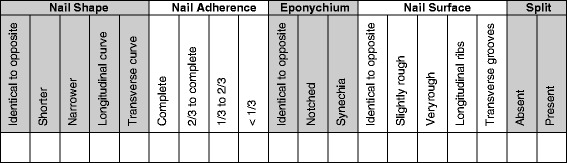
The Zook classification of fingernail appearance. All split nails, nails with less than two-thirds adherence and very rough nails are considered major deformities. Other deformities are considered minor. The sum of the minor and major variations for each fingertip are used to determine a grade of excellent, very good, fair, or poor for each result

The photograph will be assessed by a different clinician, who is blinded to the intervention the patient received. This clinician will evaluate the cosmetic appearance of the fingernail using a previously validated physician-based outcome tool developed by Zook et al. [10] (Fig. 4). Based on this scoring system, an excellent outcome for the repaired fingernail is defined as one that is identical in appearance to the same finger on the contralateral hand. A very good result exhibits one variation from identical, such as incomplete adherence, nail ridging, split nails or eponychial deformity. A good result exhibits two minor variations from identical. A poor cosmetic result exhibits more than three variations or one major variation from the same fingernail on the contralateral hand. Whilst in clinic, the patient’s parent will also be asked to rate the appearance of their child’s fingernail using a visual analogue scale (VAS). Information about any further treatment sought in between follow-up assessment time points will also be collated.

Those patients who are randomised to attend clinic at 4 months and who fail to attend for follow-up will be contacted by telephone and/or e-mail by a research nurse or clinician. These parents will be asked to email a photograph of their child’s repaired fingernail and the contralateral fingernail on the other hand for comparison as per the instructions on the email/letter sent to them. They will also be asked to e-mail their rating of the appearance of their child’s fingernail using the VAS which will be included in the e-mail.

The other half of the patients will be posted a short questionnaire asking about any further treatment sought since their 30-day assessment. A VAS will also be included for the parent and the child to rate the appearance of the fingernail. Parents will also be asked to take a photograph of the nail and send it to a secure email. These images will be collated centrally by the study team in Oxford, anonymised and sent onto independent, blinded assessors. These clinicians will be identified by the study team prior to the start of recruitment. Parents will be asked to send a digital photograph of their child’s fingernail by email to a study email account using the standardised format. An example of a standardised photograph, with the format required, will be sent to the parent for guidance.

### Blinding

NINJA-P is an open study in that those delivering the care will not be blinded to the intervention, nor the patient (and family members) who receives it. A replaced nail can take several weeks to loosen and fall off once a new nail has grown out. The only time that those involved in post-intervention assessment of the nail will be blinded is at the 4-month review. By this time, it is anticipated that all of the replaced nails will have fallen off and been replaced by a new nail. The assessment of the photographs for cosmetic appearance at 4 months will be carried out by a blinded assessor.

### Outcomes and analysis

A single analysis of data will take place once the study has ceased recruiting and the last participant has completed their final assessment. No interim analysis of data is planned nor is a statistical comparison of outcome by treatment group. Descriptive analyses of outcome data will be carried out using appropriate summary measures (e.g. number of events and percentage for binary measures). Outcome data will be grouped according to the allocated intervention irrespective of the actual treatment received. Feasibility measures will be quantified and where appropriate an associated 95 % confidence interval calculated using the Wilson Score Interval method for binary measures. All analyses will be carried out using Stata 13.0 (StataCorp. 2013. Stata: Release 13. Statistical Software. College Station, TX: StataCorp LP.). No imputation of missing data will be carried out.

Withdrawal from the study will result in exclusion of that participant’s data from analysis. If, during the course of surgery, the surgeon has to perform a procedure(s), which was part of the exclusion criteria, this will be recorded on the CRF. This is an extremely unusual event as the vast majority of these procedures (e.g. fracture fixation with a Kirschner wire, need for composite graft or nail bed graft) are predictable pre-operatively. These patients will remain in the trial.

### Ethical approval

Ethical approval for the trial was granted via Proportionate Review by National Research Ethics Service Committee, South East Cost-Surrey on the 21 January 2015. The Research Ethics Committee (REC) Reference is 15/LO/0067.

## Discussion

Nail bed injuries are the most common paediatric hand injury, yet there is a limited evidence base to guide treatment. It is common practice to replace the nail after repairing the nail bed, but this is not known to improve the outcome and may even be associated with increased post-operative complications including infection.

The results of the NINJA-P pilot study will inform the design and execution of the main NINJA trial. It will achieve this by resolving key uncertainties relating to the design of the main study, including the number of patients that present with nail bed injuries over a defined period of time, the proportion of children who agree to take part in the study, the proportion of children who then go on to receive the allocated treatment, reasons for any non-compliance and the proportion of participants with a valid response at each follow-up point. As this is a research community with limited previous involvement in clinical trials, NINJA-P will provide important practical evidence regarding the conduct of trials in this area. Potential limitations may be that the sites involved in this study may be more engaged than some of those likely to be involved in the main NINJA trial. The design of NINJA-P and the plan for the subsequent NINJA trial reflect clinical practicalities in terms of care of children requiring a nail bed repair (differences in approach between surgeons and sites) and also the limited scope of blinding attempted.

Based on this information, the NINJA trial will then fully assess whether the damaged nail should be replaced or discarded after nail bed repair in children. This large, pragmatic, multicentre, randomised, controlled study will enable the current lack of evidence regarding the treatment of this common injury to be addressed.

## Trial status

Participant enrolment commenced in April 2015 and is scheduled to be complete by the end of July 2015.

## Abbreviations

CI: chief investigator
CRF: case report form
GCP: Good Clinical Practice
NHS: National Health Service
OCTRU: Oxford clinical trials research unit
SITU: Surgical Intervention Trials Unit
